# Predicting species occurrences with habitat network models

**DOI:** 10.1002/ece3.5567

**Published:** 2019-09-04

**Authors:** Damian O. Ortiz‐Rodríguez, Antoine Guisan, Rolf Holderegger, Maarten J. van Strien

**Affiliations:** ^1^ WSL Swiss Federal Research Institute Birmensdorf Switzerland; ^2^ Planning of Landscape and Urban Systems (PLUS) Institute for Spatial and Landscape Planning ETH Zurich Zürich Switzerland; ^3^ Department of Ecology and Evolution University of Lausanne Lausanne Switzerland; ^4^ Department of Environmental Systems Science ETH Zurich Zürich Switzerland; ^5^ Institute of Earth Surface Dynamics University of Lausanne Lausanne Switzerland

**Keywords:** connectivity, cost surface, habitat network, habitat suitability, network topology, species occurrence

## Abstract

1. Biodiversity conservation requires modeling tools capable of predicting the presence or absence (i.e., occurrence‐state) of species in habitat patches. Local habitat characteristics of a patch (*lh*), the cost of traversing the landscape matrix between patches (weighted connectivity [*wc*]), and the position of the patch in the habitat network topology (*nt*) all influence occurrence‐state. Existing models are data demanding or consider only local habitat characteristics. We address these shortcomings and present a network‐based modeling approach, which aims to predict species occurrence‐state in habitat patches using readily available presence‐only records.
2. For the tree frog *Hyla arborea* in the Swiss Plateau, we delineated habitat network nodes from an ensemble habitat suitability model and used different cost surfaces to generate the edges of three networks: one limited only by dispersal distance (Uniform), another incorporating traffic, and a third based on inverse habitat suitability. For each network, we calculated explanatory variables representing the three categories (*lh*, *wc*, and *nt*). The response variable, occurrence‐state, was parametrized by a sampling intensity procedure assessing observations of comparable species over a threshold of patch visits. The explanatory variables from the three networks and an additional non‐topological model were related to the response variable with boosted regression trees.
3. The habitat network models had a similar fit; they all outperformed the non‐topological model. Habitat suitability index (*lh*) was the most important predictor in all networks, followed by third‐order neighborhood (*nt*). Patch size (*lh*) was unimportant in all three networks.
4. We found that topological variables of habitat networks are relevant for the prediction of species occurrence‐state, a step‐forward from models considering only local habitat characteristics. For any habitat patch, occurrence‐state is most prominently influenced by its habitat suitability and then by the number of patches in a wide neighborhood. Our approach is generic and can be applied to multiple species in different habitats.

## Graphical abstract

We present a network‐based modeling approach to predict species presence or absence (occurrence‐state) in habitat patches. The development includes a method to derive absences from unsystematically sampled presence‐only data and was proved effective on data from the tree frog *Hyla arborea*. Our approach is generic and designed to work with widely available data, so it can potentially find use in a variety of environments and for multiple species.

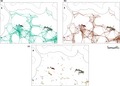

## 1 INTRODUCTION

Knowledge about the spatial distribution of species is a key element for any conservation effort. To gain insights on the presence or absence of a species at specific locations (occurrence‐state; from occurrence in Kéry & Schaub, 2012), it is necessary but not sufficient to consider the conditions that make the specific sites suitable for a species, that is, to define patches of suitable habitats (sensu Guisan & Zimmermann, 2000). One also needs to take into account habitat connectivity, which is the way the suitable habitat patches are accessible and thus connected to each other, or “the degree to which the landscape facilitates or impedes” the movement of species (Taylor, Fahrig, Henein, & Merriam, 1993). The consideration of connectivity is important, as a habitat patch in which the environmental conditions are suitable for a certain species can actually be unoccupied due to the inability or low probability of the species to reach the patch (Barve et al., 2011). In such a case, the occurrence‐state of the known suitable habitat patch would be 0, as occurrence‐state is a property of habitat patches with two alternative states: presence (1) or absence (0). To better capture the factors influencing the occurrence‐state of a species, and to be able to make predictions about this state, it is necessary to develop new modeling approaches that do not only consider the local conditions in a habitat patch, but also the connectivity between patches. This was the main goal of the present study.

Habitat patches and their connectivity can be represented in a network‐theoretical framework. Since the work of Bunn, Urban, and Keitt (2000), spatially explicit habitat network models have been in common use (e.g., Duflot, Avon, Roche, & Bergès, 2018; Saura & Pascual‐Hortal, 2007; Urban, Minor, Treml, & Schick, 2009). In such networks, nodes usually represent habitat patches potentially inhabited by a species, and edges commonly represent potential movement among them. In many habitat networks, edges are modeled with cost surfaces (i.e., raster maps in which each cell has a value of resistance to movement) from which likely movement routes can be derived (Adriaensen et al., 2003; McRae, 2006). In other cases, edges are modeled with straight‐line transects (Jordán, Magura, Tóthmérész, Vasas, & Ködöböcz, 2007; van Strien et al., 2014). The specific arrangement of nodes and edges is the network topology (Kauffman, 1993; Urban et al., 2009), which can be analyzed at different scales, ranging from the immediate vicinity of a patch to the whole network (Baranyi, Saura, Podani, & Jordán, 2011). Following this logic, the presence of a species in a certain habitat patch is influenced by three different key categories of factors, which can be summarized with the following conceptual equation:

(1) $$\psi_{i}=f\left({\mathrm{lh}}_{i},{\mathrm{wc}}_{i},{\mathrm{nt}}_{i}\right)$$

where *ψ_i_* is the occurrence‐state of a species (whether it is present or absent) in a habitat patch *i*, *lh* *_i_* refers to the local habitat characteristics of such patch, *wc* *_i_* is the weighted connectivity of the patch to surrounding patches, and *nt* *_i_* is the place of the patch (node) in the network topology.

Local habitat characteristics (*lh* *_i_*) are defined by the properties of suitability and size of a habitat patch. Patch size is an important factor in metapopulation biology (Hanski, 1992), and its relevance is widely acknowledged in studies dealing with occurrence and distribution of species (Hodgson, Moilanen, Wintle, & Thomas, 2011; Saura & Pascual‐Hortal, 2007). The suitability of a patch is determined by the environmental requirements (i.e., environmental niche) of species. These requirements can be assessed with habitat suitability modeling (HSM), which aims to predict the distribution of species across a study area based on mapped environmental factors (Guisan & Zimmermann, 2000; Thuiller & Münkemüller, 2010).

Habitat connectivity depends on the movement ability and behavior of a species, reflected in species‐specific maximum dispersal distances (Jenkins et al., 2007). It also depends on factors that facilitate or inhibit the movement of a species through the landscape between neighboring suitable patches (Prevedello & Vieira, 2009). The weighted connectivity (*wc*) component of conceptual Equation (1) includes those variables that explicitly incorporate the probability of traversing the landscape matrix (the latter determining the “weights”) into their calculation. The *wc* factors give rise to the emergent large‐scale structure of a network. The network topology (*nt*) refers to this large‐scale structure (Albert & Barabási, 2000). For a given node *i*, *nt* *_i_* refers to variables that describe its neighborhood, position, and importance in the whole network, independent of any weights specific to a certain environmental or species‐specific context. The context‐independent nature of *nt* variables makes them ideal to compare habitat networks of different species in different environments, as well as to compare habitat networks with other kinds of natural networks (Watts & Strogatz, 1998).

Determining the occurrence‐state of a habitat patch is difficult for non‐sessile species (MacKenzie et al., 2002). Although it can be performed by site occupancy models (Kéry & Schaub, 2012), these can only be used in situations where sites have been sampled in a regular and systematic way (MacKenzie et al., 2006). Another difficulty is the empirical estimation of connectivity between habitat patches, which is usually performed by means of mark–recapture, radio tracking, GPS sensors, or genetic methods (Kool, Moilanen, & Treml, 2013; Straka, Paule, Ionescu, Štofík, & Adamec, 2012). Due to their high costs and labor intensity, these methods are usually not implemented over large spatial scales, for several species, or by institutions under economic hardship. In summary, the determination of both the occurrence‐state of habitat patches and of the connectivity among them is based on data that are relatively expensive, laborious, and time‐consuming to obtain. In contrast, spatially explicit data on species observations are readily available in many countries, such as the data aggregated in the GBIF international database (http://www.gbif.org) or in the Swiss InfoSpecies database (http://www.infospecies.ch). These data consist of confirmed presences, but usually do neither contain any absences nor information on whether all potential habitat patches were surveyed. Given these biases, it is a challenge to parameterize habitat networks with such incomplete data. Nevertheless, given the high prevalence of such data, it is worthwhile to explore the possibilities of using it to parametrize habitat network models aiming to predict species occurrence. By aggregating observation data from groups of comparable species (Anderson, 2003) to determine a habitat patch's sampling intensity, we expect that likely absences for a focal species can be estimated.

In this study, we developed a habitat network modeling approach to predict species occurrences in habitat patches following conceptual Equation (1). We aimed to develop a generic method that (a) includes insights about the topology of the habitat networks and (b) makes use of readily available presence‐only records. We expected that the incorporation of network topological variables would increase the explanatory power of models as compared to nontopological ones, addressing the omission of connectivity factors incurred by traditional models capable of predicting species occurrences (such as HSM and resource selection models; Boyce, Vernier, Nielsen, & Schmiegelow, 2002). We anticipate that the approach can be applied to a multitude of species in different environments at minimal cost. We exemplify our approach with the European tree frog (*Hyla arborea* L.) in the Swiss Plateau.

**Figure 1:**
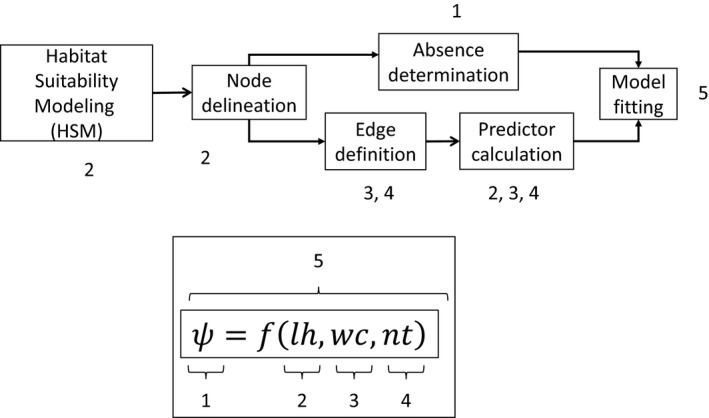
Workflow of steps used in this study. The numbers indicate the relation of each step to Equation (1) (inset below)

We followed a multistep procedure with two modeling stages (Figure 1). First, we used HSM to delineate suitable patches, that is, the nodes of the network. We then defined the edges based on least‐cost calculations on different cost surfaces, which incorporated different environmental, biological, and human influences on the landscape, generating three different networks. From these networks, we calculated several variables quantifying the three categories of factors (i.e., lh, wc, and nt) in Equation (1), which were used as explanatory variables in models that related them to the response variable occurrence‐state. We then compared the fit of models with and without the *wc* and *nt* variables. In order to calculate occurrence‐state, we developed an approach inspired by Anderson (2003) that uses comparative sampling intensity to define absences of the focal species in habitat patches. Finally, by means of boosted regression trees (BRTs), we tested the explanatory power of predictor variables related to the three factors of Equation (1) on occurrence‐state.

## 2 METHODS

### 2.1 Study area, focal species and presence records

**Figure 2:**
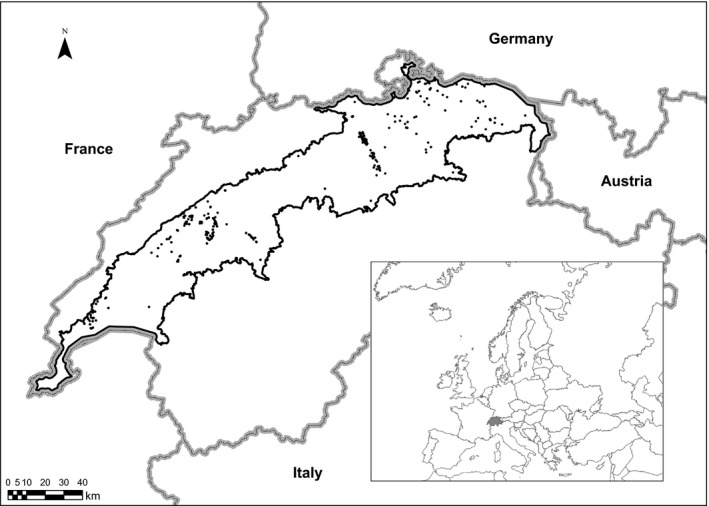
Location of the study area (Swiss Plateau; black line) in Switzerland (gray line, solid gray in inset) and presence records of *Hyla arborea* (black dots)

Our study area consisted of the Swiss Plateau, a densely populated region (426 inhabitants/km^2^; Müller‐Jentsch, 2012) of 11,168 km^2^, where strong increases in landscape fragmentation and urban sprawl have recently occurred (Roth, Schwick, & Spichtig, 2010). The area is dominated by human land use, with a patchy distribution of settlements, agricultural land, and forests. The exact shape of the study area (Figure 2) was defined by the boundaries of the Swiss Plateau from the official map of the biogeographical regions of Switzerland (OFEV, 2011) minus a 2 km (i.e., commonly reported amphibian dispersal distances; Smith & Green, 2005) negative buffer away from the international borders of Switzerland to prevent border effects. We chose the European tree frog (*H. arborea* L.) as our focal species, as it is a neither abundant nor rare habitat specialist, vulnerable to environmental disturbances and restricted to well‐defined natural features, which are areas close to sunny forest edges and bushy landscape elements surrounding vegetation‐poor ponds, in which it spawns (Clauzel, Girardet, & Foltete, 2013).

Our dataset on species occurrences consisted of geopositioned records of 13 amphibian species (Appendix A1) that were sampled from water bodies (mainly ponds, but also shallow lakeshores) between 2006 and 2015 across the Swiss Plateau, provided by InfoSpecies‐KARCH (http://www.infospecies.ch). The records in this dataset originated from a variety of sources and observers and are limited to sites visited and reported on during the above‐mentioned period. Some sites were visited only once, while others were visited annually or many times. It was also not clear whether observers reported all species encountered at a visit, an arbitrary subset of species or a single species. Absences of particular species from particular sites were thus not explicitly reported. Such data limitations are frequently encountered in national and international observation databases. Records on amphibian occurrences were aggregated at a 1 ha resolution. In total, the dataset consisted of 2,354 locations with at least one amphibian species presence in one or more years. Out of these, 291 contained the focal species *H. arborea* (Figure 2).

### 2.2 Habitat suitability modelling

For HSM, we compiled a dataset of 25 environmental predictor variables based on previous studies describing the environmental preferences of pond‐based amphibians in general and *H. arborea* in particular (Pellet, Hoehn, & Perrin, 2004; Van Buskirk, 2005; Zanini, Pellet, & Schmidt, 2009), as well as additional variables quantifying human influence on ecosystems. Our final HSM predictors fell under three basic categories: human influence, natural landscape features, and climate variables. All predictor variables were converted to a resolution of 1 ha. Circular moving windows with a 2 km radius (common dispersal distance of amphibians) were used for calculating many of the predictors. We eliminated collinear predictor variables based on pairwise Pearson's correlation coefficients (Gillham, 2001) with a reference threshold of 0.75 and based on variance inflation factors (VIF) with a threshold of 0.9, using the packages USDM (Naimi, Hamm, Groen, Skidmore, & Toxopeus, 2014) and stats in R 3.3 (R Development Core Team, 2016). The removed variables were mean annual precipitation, total noise at daytime, recreation intensity, highway density, and density of roads. This led to a final selection of 20 predictor variables for HSM (Appendix A2 lists the HSM predictors; Appendix A3 gives a short description and the sources of the data). All data processing was carried out with ArcGIS 10.4.1. (ESRI, 2016) in Python 2.7 (Python Software Foundation, 1995).

In order to delineate potential habitat patches of *H. arborea*, we generated an ensemble habitat suitability model (HSm) in which the 291 presences of *H. arborea* constituted the response variable. To prevent pseudoreplication, we included only one record of *H. arborea* per sampling site, even if the species was observed in multiple years. We generated 10,000 pseudoabsences as recommended by Barbet‐Massin, Jiguet, Albert, and Thuiller (2012), with one round of pseudoabsence selection. We developed an ensemble using the R‐package Biomod2 (Thuiller, Georges, Engler, & Breiner, 2016), which does multiple runs of different models, projects the models spatially, and generates consensus projections between the different models. In this study, we used the mean ensemble of a generalized linear (GLM), a random forest (RF), and a maximum entropy (MaxEnt) model. The models were evaluated with ROC AUC, with a quality threshold of AUC ≥ 0.7 (Bulluck, Fleishman, Betrus, & Blair, 2006). To binarize the continuous habitat suitability maps, the applied criterion was the point in the ROC curve that minimizes the difference between sensitivity and specificity. We used default settings unless otherwise specified.

### 2.3 Node delineation

**Figure 3:**
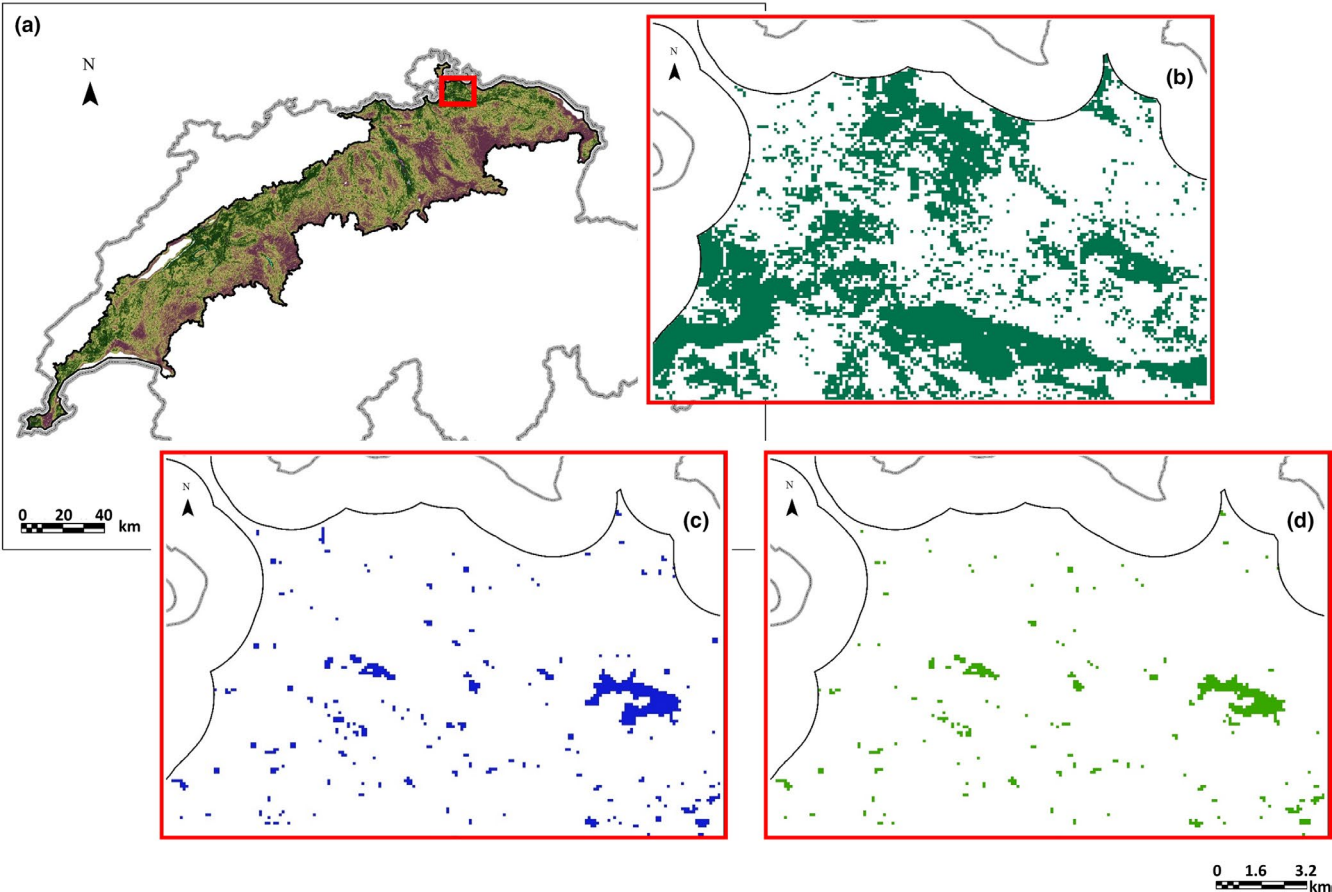
Continuous (a) and binary (b; close‐up of red area in a) suitability maps yielded by the ensemble habitat suitability model for *Hyla arborea* in the Swiss Plateau. Discrete habitat patches (d; same close‐up as in b) were produced by the application of a mask (c; same close‐up as in b)

HSM resulted in a map indicating where the environmental conditions were potentially suitable for *H. arborea*. In order to delimit suitable habitat patches for this species, we intersected the binary results of the ensemble HSm with those areas in which *H. arborea* can reproduce, namely water bodies in the Swiss Plateau. Water bodies were defined by merging several spatial datasets: lakeshores (Swisstopo, 2016), mires (OFEV, 2010), amphibian spawning sites (BAFU, 2016), and all locations with at least one occurrence of at least one amphibian species in the period 2006–2015. The merged layer constituted a mask that was overlaid with the binarized HSm (Guisan et al., 2006; Figure 3). A habitat patch was considered unique if it was not connected to any other patch under a Moore neighborhood criterion (i.e., considering all eight neighbors of a raster cell). For each patch, we determined its size (ha) and its mean habitat suitability, which were later used as explanatory variables for the occurrence‐state modeling (see below). The identified habitat patches constituted the nodes of the habitat network.

### 2.4 Edge definition

Between pairs of nodes, we defined edges based on least‐cost calculations (Etherington, 2016). We developed an algorithm that takes as input a binary raster of habitat patches and a cost surface. The algorithm determines the least cumulative cost between patches and draws an edge between patches if the total cost is below a certain threshold. Translation of dispersal probabilities into dispersal costs and vice versa was performed following the p2p function of the R‐package PopGenReport (Adamack & Gruber, 2014):

(2) $$\mathrm{cost}=\log\left(\mathrm{prob}\right)/\log\left(p\right)*d0$$

in which *d*0 is the dispersal distance of a proportion *p* of individuals, *prob* is the probability of dispersal between patches, and *cost* is the cost–value associated with a certain probability *prob*. We set *p = *.5, so that *d*0 equaled the median dispersal distance. We set the dispersal probability threshold beyond which no edges were drawn to 0.0001. Subsequently, *d*0 was set to 200 m so that no edges were formed over cost distances of 2,658. When cost distances are just Euclidian distances, 2,658 m is slightly above the reported average maximum dispersal distance of *H. arborea* (Clauzel et al., 2013).

Making use of three different cost surfaces, we created three different networks: a Uniform, a Traffic, and an Inverse Habitat Suitability network. In the Uniform network, the cost distance was equal to the Euclidean distance among habitat patches. In the Traffic network, the default cost–value of a raster cell was 1, and for all the raster cells that intersected with a road (excluding tunnels), the traffic intensity on the respective road was converted to a cost–value. To do this, we calculated the probability that an animal successfully crosses a road according to van Langevelde and Jaarsma (2009, equation A1.4). These authors include speed and physical dimensions of vehicles and animals, as well as traffic volumes on intersecting roads, to calculate probabilities of successful road crossing. The parameter settings for *H. arborea* were taken from Van Strien and Grêt‐Regamey (2016). Subsequently, the calculated probabilities were transformed to costs using Equation (2). The traffic values per road segment were those calculated by the 2010 version of the Swiss national passenger transport model (ARE, 2010). For the Inverse Habitat Suitability network (HabSuit), we assumed that the probabilities of dispersing through the most unsuitable and suitable terrain were 0 and 1, respectively. Therefore, the continuous habitat suitability raster from HSM was divided by the maximum suitability and the inverse value was then taken as cost–value (sensu Ziółkowska, Ostapowicz, Radeloff, & Kuemmerle, 2014). With this approach, not only the network topology differed between the cost surfaces, but also the weight of individual edges in the network. Edge weights were calculated by transforming the least‐cost values to dispersal probabilities following Equation (2); hence, costlier paths have lower dispersal probabilities. Edge calculations were performed using the Python packages numpy (Oliphant, 2006), arcpy (ESRI, 2016), and igraph (Csardi & Nepusz, 2006).

### 2.5 Calculation of explanatory variables for occurrence‐state modeling

We prepared a set of explanatory variables for network model assessment, which quantified the three types of factors from Equation (1) as patch (node) properties. To address the aspect of network topology (*nt* *_i_*) at different scales, we calculated for each habitat patch the degree, third‐order neighborhood, and betweenness centrality. The degree is the number of connections (edges) a specific node *i* has to other nodes (Jordán & Scheuring, 2004). The third‐order neighborhood measures the number of nodes (patches) that can be reached in maximally three topological steps through the network (Csardi & Nepusz, 2006). To measure the influence of topology at the whole‐network scale, we used betweenness centrality, which measures how many connections between all node pairs in the network pass‐through node *i* (Freeman, 1978). While Baranyi et al. (2011) define it as a meso‐scale measure, it is actually calculated considering all other nodes in the network, so it is an appropriate proxy to check how the whole‐network structure affects a node‐specific property.

To account for the weighted connectivity of patches (*wc* *_i_*), the calculated variable was the strength, which is the sum of the weights of all the edges connecting a node to others (Barrat, Barthélemy, Pastor‐Satorras, & Vespignani, 2004). It is thus also considered a topological variable. We also calculated the habitat availability, which is a hybrid variable incorporating aspects of *nt* and *lh*. This measure calculates a weighted sum of all patch sizes that can be reached from a focal patch *i*. The weights are calculated as the maximum product probability between two patches. Habitat availability is similar to the probability of connectivity index of Saura and Pascual‐Hortal (2007), with the main difference that it is calculated for each node separately (not summed over all nodes) and not divided by the total habitat area. In order to achieve efficient computation times, we limited the process to consider patches only up to second‐order neighborhood, after having observed only negligible change for higher neighborhood order values.

In addition to those network topological variables, we also included the size (ha) and the mean habitat suitability (habitat suitability index; HSI) values of the patches to evaluate the influence of local habitat characteristics (*lh* *_i_*). The habitat suitability values per patch were obtained by calculating the mean habitat suitability of all pixels that made up a discrete patch. The size was an attribute generated when defining the discrete patches.

### 2.6 Determination of absences of *H. arborea*

To define the absence values of the binary response variable occurrence‐state*,* we used an adapted version of the approach used by Anderson (2003), based on comparative sampling intensity. For each habitat patch, we assumed that a presence record of any amphibian species in a particular year represented a confirmed visit to the patch in that year by an observer familiar with amphibians. Furthermore, we assumed that if a patch was visited a considerable number of years and a certain focal species was not reported, it is likely that such species was indeed not present in that patch during these years. We calculated the total number of times a patch had been visited (*V_t_*) by aggregating all observations of the 13 amphibian species in the data set (Appendix A1). Multiple amphibian species recorded in one particular year for a certain location were counted as only one visit. As we had 10 years of observations with a temporal resolution of 1 year, the maximum number of visits (*V_t_*) was 10. For each patch, we also calculated the number of times *H. arborea* was observed (*V*_h_), and for each *V_t_* value, we calculated the mean *V*_h_. We assigned a likely absence (occurrence‐state = 0) to all those patches with a *V_t_* value that corresponded to a mean *V*_h_ ≥ 1 (i.e., on average *H. arborea* was found at least once over the years) and in which *H. arborea* had not been recorded. Patches that neither contained a confirmed presence (from the original occurrence data on *H. arborea*) nor a likely absence had an unknown occurrence‐state and were therefore excluded from subsequent analyses.

### 2.7 Occurrence‐state network model fitting

In order to test what kind of variables were most important for explaining the occurrence‐state of a species in a habitat patch, we ran boosted regression trees (BRTs; Elith & Leathwick, 2017). This modeling technique has been proven useful for the analysis of complex ecological data. It can handle interactions among variables and nonparametric relationships, and integrates the calculation of variable importance (Elith, Leathwick, & Hastie, 2008). The three different habitat networks (Uniform, Traffic, and HabSuit) were used to build three separate models. In all of them, we used the seven explanatory variables described above. In addition, we built a model without topological variables (noTopo), for which the only explanatory variables were patch size and HSI. For all four models (Summarized in Table 1), the response variable was the occurrence‐state of *H. arborea*.

**Table 1:** Summary of the three network‐based models (Uniform, Traffic, and HabSuit) and the model without topological predictors fitted by BRT's

Model	Resistance surface used to define edges	Predictors included
Uniform	Cost–value equal to Euclidean distance among habitat patches; edge formation limited by dispersal distance only.	Degree Strength Third‐order neighborhood Habitat availability Betweenness centrality Mean HSI Mean patch area
Traffic	Traffic intensity on intersecting roads converted to cost–value.	Degree Strength Third‐order neighborhood Habitat availability Betweenness centrality Mean HSI Mean patch area
HabSuit	Cost–value defined by inverse of maximum‐weighted habitat suitability index.	Degree Strength Third‐order neighborhood Habitat availability Betweenness centrality Mean HSI Mean patch area
noTopo	None. Network topology not considered.	Mean HSI Mean patch area

BRT models are based on an aggregation of numerous classification trees (at least 1,000 as recommended by Elith et al., 2008). The learning rate (lr) and tree complexity (tc) influence the number of trees that are used in a final BRT model. As stochastic factors give rise to differences in the prediction each time the model is run, we used 100 runs with lr = 0.001, tc = 5, and a bagging fraction of 0.75 (following the general guidelines of Elith et al., 2008) with the gbm.step function of the R‐package dismo (Hijmans, Phillips, Leathwick, & Elith, 2017). This function searches for the number of trees that yields the lowest deviance. We evaluated the predictive power of the models by comparing the distribution of their cross‐validated AUC values (cv‐AUC) over the 100 runs. We assessed the different variables with the distributions of their importance scores over all 100 runs.

## 3 RESULTS

### 3.1 Habitat suitability modeling and patch delineation

The ensemble HSm, a mean of nine models**,** yielded a continuous and a binarized habitat suitability map (Figure 3a,b). The mask (Figure 3c) applied on the binarized suitability map yielded the definitive delineation of habitat patches (Figure 3d). The total number of habitat patches was 1900.

### 3.2 Edge definition

**Figure 4:**
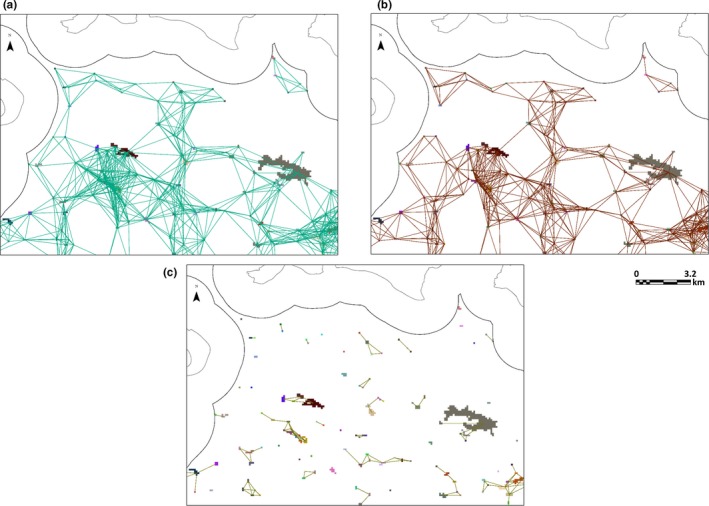
Habitat networks of *Hyla arborea* in the Swiss Plateau (same close‐up as in Figure 3). The networks were based on three different cost surfaces: Uniform (a), Traffic (b), and Inverse Habitat Suitability (HabSuit; c)

The three different cost surfaces yielded different networks. The Traffic network comprised 254 components (groups of linked patches) and the Uniform network 134 components. The HabSuit network was the most sparsely connected, divided into 850 components (Figure 4).

### 3.3 Values of explanatory variables

Mean values of all variables are shown in Table 2. For most network variables, the HabSuit network exhibited much smaller values (Table 2) than the Uniform and Traffic networks. For habitat availability, the differences were less pronounced.

**Table 2:** Mean values of explanatory variables for the three different networks of *Hyla arborea* in the Swiss Plateau: Uniform, Traffic, and Inverse Habitat Suitability (HabSuit)

	Uniform	Traffic	HabSuit
Degree	11.32	8.86	4.02
Strength	0.58	0.49	0.22
Third‐order neighborhood	41.63	30.39	11.65
Habitat availability	311,578.5	297,289.4	265,234.7
Betweenness centrality	1,029.20	911.85	34.19
Mean HSI	650.15	650.15	650.15
Mean patch area (ha)	18.97	18.97	18.97

### 3.4 Determination of absences

**Figure 5:**
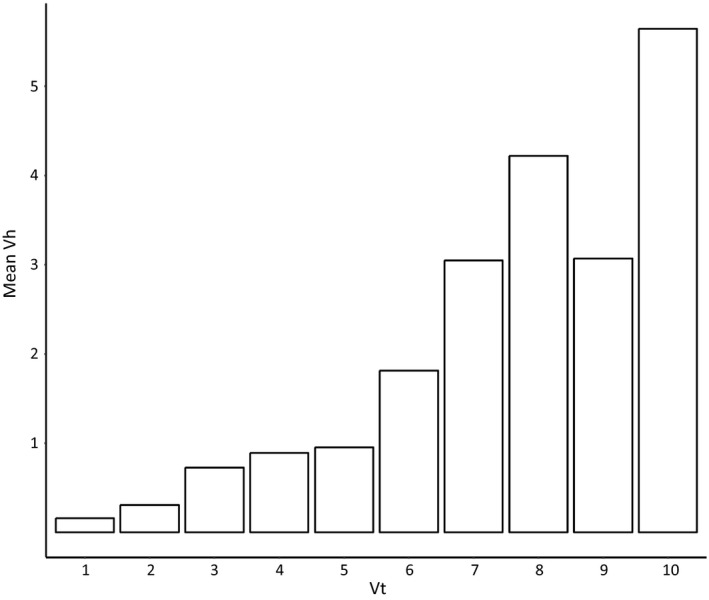
Relationship between the number of times a patch has been visited (*V_t_*) in the period 2006–2015 and the mean number of times that *Hyla arborea* has been spotted for any number of visits (mean *V*_h_) in the Swiss Plateau

The mean number of times that *H. arborea* was sighted (mean *V*_h_) increased with the number of times a patch was visited (*V_t_*). At *V_t_* ≥ 6, mean *V*_h_ ≥ 1.8125, indicating that on average there was more than one sighting of *H. arborea* when a site was visited six or more times (Figure 5). Therefore, we regarded every patch with *V_t_* ≥ 6 and no confirmed *H. arborea* sighting as a likely absence. In doing so, we determined 46 likely absences of *H. arborea*, complementing the 209 confirmed presences.

### 3.5 Occurrence‐state network model fitting

**Figure 6:**
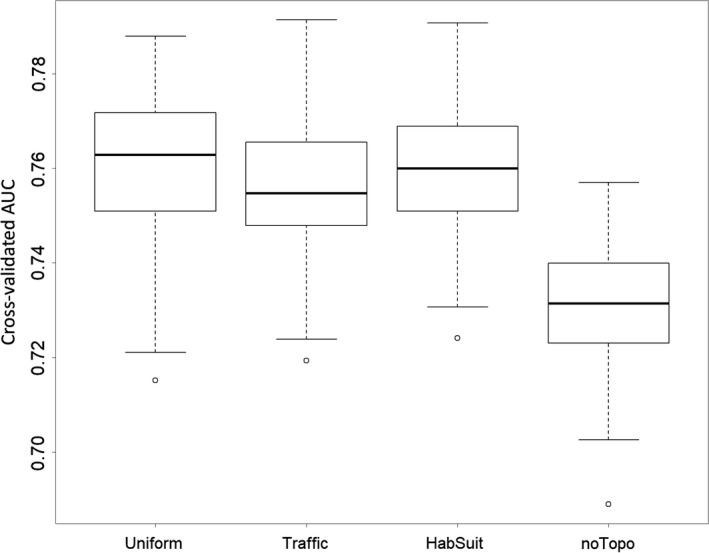
Distributions of cross‐validated AUC scores over 100 runs for four models (Uniform, Traffic, and HabSuit networks, and noTopo) for *Hyla arborea* occurrence‐state in the Swiss Plateau

The boosted regression trees showed a similar predictive performance among the three network models, as indicated by the distributions of their cv‐AUC scores (Figure 6). The model without topological measures (noTopo) was outperformed by all three models with topological variables (Table 3).

**Table 3:** Mean cross‐validated AUC scores over 100 runs of four models (Uniform, Traffic, HabSuit, and noTopo) for *Hyla arborea* occurrence‐state in the Swiss Plateau

	Uniform	Traffic	HabSuit	noTopo
Cross‐validated AUC score	0.7610	0.7552	0.7597	0.7309

HSI was the variable with the highest importance in all models. While the lowest mean HSI value in a suitable patch determined by the ensemble HSm was 382, the partial dependence plots for all models (Appendix A4) pointed toward a threshold HSI value above 500. In all the network models, third‐order neighborhood was consistently the second most important variable (importance above 13%), followed by strength in the Uniform and Traffic models, and by habitat availability in the HabSuit model. Patch size was consistently among the least important variables in all models (Table 4).

**Table 4:** Mean relative importance (in %) of seven predictors over 100 runs of boosted regression trees (BRTs) for four models (Uniform, Traffic, HabSuit, and noTopo) for occurrence‐state of *Hyla arborea* in the Swiss Plateau

Explanatory variable	Mean relative importance			
	Uniform	Traffic	HabSuit	noTopo
Habitat Suitability Index (HSI)	43.10	44.96	53.67	84.34
Third‐order neighborhood	13.05	19.49	19.80	–
Strength	12.86	11.25	5.87	–
Habitat availability	10.01	9.08	13.26	–
Degree	8.46	5.55	0.33	–
Betweenness centrality	7.69	3.77	0.79	–
Patch area	4.82	5.90	6.27	15.66

## 4 DISCUSSION

The goal of this study was to develop an approach to assess the occurrence‐state of a species in habitat patches, one which would be inexpensive and practical by using widely available species presence data, and which would have an added predictive value by incorporating topological properties of the species' habitat networks as predictor variables. Our results support the expectation that topological variables of habitat networks are indeed relevant for explaining and predicting the occurrence‐state of a species in habitat patches. This is showcased by the results on BRT model comparison, in which the model without topological variables (noTopo) had the poorest performance in terms of its mean cv‐AUC score (Figure 6).

Following our other main objective, a novel aspect we present in this study is the derivation of likely absences from presence‐only data. Given that we use unsystematically collected data with low temporal resolution, it was not possible to use traditional site occupancy models (Kéry & Schaub, 2012). Instead, we developed a comparative sampling intensity approach, which yielded the likely absences necessary to model occurrence‐state. A drawback of this approach was that we could only define likely absences for a small fraction of the habitat patches originally defined: our BRT models were built on 255 out of 1,900 patches, and the final response variable only included 46 absences. Nevertheless, even with this relatively small dataset, all of the network models had mean cv‐AUC scores above the 0.75 AUC threshold of acceptability for good models (Elith, 2002).

The difference in predictive power between the three different network models was slight. While the Uniform network had a better mean predictive performance than the others did (Table 3), the difference was too small to warrant any conclusions regarding a most probable movement hypothesis for the focal species *H. arborea*. In order to study this aspect, it may be worthwhile to experiment with different combinations of cost factors or ways to define them (such as nonlinear cost increase; Duflot et al., 2018). The application of the method to ecologically different taxa and landscapes of various sizes might reveal greater differences among network models. The contrast between different models as presented here could be used to identify the most likely movement hypothesis for a given target species and therefore indicate the optimal kinds of network models to use in other ecological contexts.

The explanatory variable with the highest importance across all three network models was the mean habitat suitability index per patch (HSI). This was partially expected, as the baseline requirement for occupancy of a patch is to be suitable habitat. However, the values of HSI related to presence or absence had a different threshold than the one from the initial habitat suitability model (see partial dependence plots, Appendix A4). This was probably due to the contrast of confirmed presences with an informed selection of likely absences instead of random pseudoabsences as in HSM. The approach to determine likely absences applied here could thus be used to fine‐tune binarization thresholds of habitat suitability models in other applications.

The consistent importance of the third‐order neighborhood variable shows that the number of patches at this wider scale influences the occurrence‐state of a species more than in the immediate vicinity (as shown by the lower importance of degree). The centrality of a patch at the whole‐network scale did not prove to be especially relevant for the occupancy‐state of a species. Another variable that was not important in any of the models was patch area. This arrangement of variable performances shows the complementarity of the different factors of Equation (1) in determining species occurrence‐state. Local habitat characteristics are important, but offer an incomplete assessment, which can be enhanced by the incorporation of topological variables.

Our multistep approach of occurrence‐state assessment thus achieved the expectation of improving predictive performance with the integration of connectivity and network topological considerations. Methods that incorporate these considerations should lead to better‐targeted conservation actions, with more satisfactory outcomes. Our approach is generic and can be applied to any other wildlife species. In addition to presence observations of the focal and related species, the main data requirements for its application are spatial datasets of variables deemed important for the habitat suitability (for patch delineation) or connectivity (for edge definition) of the focal species. Patch delineation, easier for habitat specialists such as *H. arborea* (Van Buskirk, 2011), can be helped by the implementation of masks (e.g., excluding roads) to reduce patch size for more generalist species.

Further testing of our approach will indicate how widely it can be implemented, and ground‐truthing likely absences would be particularly interesting. Although confirming absences is a difficult endeavor, novel techniques like eDNA (Deiner et al., 2017) could help with this task. An interesting expansion of our approach would be to incorporate temporal dynamics. With a higher temporal resolution (we only had 10 time points), one could get further insights into how occurrences in one time step influence those in the next, thereby incorporating perspectives from metapopulation theory (Hanski, 1998) or research on dynamical complex networks in other disciplines (e.g., Alvarez‐Buylla et al., 2007; Sinatra, Wang, Deville, Song, & Barabási, 2016). Our approach also opens the possibility for multi‐species analyses, comparing the networks of different taxa. With such an analysis, it would become possible to identify key spatial elements across multiple networks and areas of strategic importance for the conservation of groups of species (Foltête, 2019). Our approach is fully expandable, and we hope it can find use in conservation management in different contexts around the world, often in dire need of effective and inexpensive methods.

## CONFLICT OF INTERESTS

None declared.

## AUTHOR CONTRIBUTIONS

All authors conceived the ideas and designed the methodology. DOR analyzed the data and wrote the manuscript. RH, AG, and MvS reviewed and commented critically on the manuscript. All authors gave final approval for publication.

### OPEN RESEARCH BADGES

This article has earned an Open Data Badge for making publicly available the digitally‐shareable data necessary to reproduce the reported results. The data is available at https://doi.org/10.5061/dryad.sc818d5.

## Data Availability

The scripts used to develop the method are available from the Dryad Digital Repository (https://doi.org/10.5061/dryad.sc818d5). The original data are either conservation‐relevant or property of Swiss public institutions, only available by independent agreements with them; therefore, it is not included in the Dryad data package of this article. Upon request, the authors can give indications on how to request access to the data from the relevant institutions.

## Appendix A1

The 13 amphibian species of which presence records in the Swiss Plateau were used in this study

**Table:**

Species	Common name
*Hyla arborea*	European Tree Frog
*Alytes obstetricans*	Midwife Toad
*Bombina variegata*	Yellow‐bellied Toad
*Bufo bufo*	Common Toad
*Epidalea calamita*	Natterjack Toad
*Ichthyosaura alpestris*	Alpine Newt
*Lissotriton helveticus*	Palmate Newt
*Pelophylax* sp. *(P. lessonae + P. esculentus)*	Green Frog complex (Pool Frog and Edible Frog)
*Pelophylax ridibundus*	Lake Frog
*Rana dalmatina*	Agile Frog
*Rana temporaria*	Grass Frog
*Triturus carnifex*	Italian crested newt
*Triturus cristatus*	Northern crested Newt

## Appendix A2

Final set of 20 predictor variables used in habitat suitability modelling (at a resolution of 1 ha), classified in three main categories

**Table:**

Category	Predictor	Variable type
Human influence	Density of traffic	Continuous
	Density of railways	Continuous
	Total noise at nighttime	Continuous
	Population density	Continuous
	Agriculture density	Continuous
	Arable land	Binary
	Green settlements	Binary
	Grey settlements	Binary
	Meadows and farm pastures	Binary
	Orchards, vineyards, horticulture	Binary
Natural landscape features	Deciduous forest coverage	Binary
	Mixed forest coverage	Binary
	Coniferous forest coverage	Binary
	Density of forest (general)	Continuous
	Distance to forest edge	Continuous
	Presence of rivers	Binary
	Slope	Continuous
Climatic variables	Mean summer precipitation	Continuous
	Mean annual direct solar radiation	Continuous
	Mean annual temperature	Continuous

## Appendix A3

Predictor variables in habitat suitability modeling, their content and source

**Table:**

Predictor	Content	Source
Density of traffic	Individual vehicle traffic for 2010	NPVM with tunnels removed (ARE, 2010)
Density of railways	Density of rail network	SwissTLM3D (Swisstopo, 2016)
Total noise at nighttime	Nighttime rail noise combined with nighttime street noise	EMPA (2011)
Population density	Statistics on Swiss population, geolocated	STATPOP (BFS, 2015)
Agriculture density	Density of agricultural areas, derived from an aggregate of the four main categories of agricultural land use	Arealstatistik (OFS, 2010)
Arable land	Agricultural area taken from point estimates on 72 land use categories	Arealstatistik (OFS, 2010)
Green settlements	Area of green spaces in settlements taken from point estimates of 72 land use categories	Arealstatistik (OFS, 2010)
Grey settlements	Area of grey (sealed areas and buildings) areas taken from 72 land use categories	Arealstatistik (OFS, 2010)
Meadows and pastures	Area of meadows and pastures taken from point estimates of 72 land use categories	Arealstatistik (OFS, 2010)
Orchards, vineyards, horticulture	Area of orchards, vineyards and horticulture taken from point estimates of 72 land use	Arealstatistik (OFS, 2010)
Deciduous forest coverage	Occurrence of deciduous forests	Waldmischungsgrad (BFS, 2013)
Mixed forest coverage	Occurrence of mixed forests	Waldmischungsgrad (BFS, 2013)
Coniferous forest coverage	Occurrence of coniferous forests	Waldmischungsgrad (BFS, 2013)
Density of forest	Density of all forest types of Switzerland	Waldmischungsgrad (BFS, 2013)
Distance to forest edge	Distance to forest edges	Waldmischungsgrad (BFS, 2013)
Presence of rivers	Presence of rivers	SwissTLM3D (Swisstopo, 2016)
Slope	Calculated from a digital elevation model	swissALTI3D (Swisstopo, 2018)
Mean summer precipitation	Mean summer precipitation (1961–1990)	Broennimann, Randin, Zimmermann, and Guisan (2003)
Mean annual direct solar radiation	Mean annual direct solar radiation (1961–1990)	Broennimann et al. (2003)
Mean annual temperature	Mean annual temperature (1961–1990)	Broennimann et al. (2003)

## Appendix A4

Partial dependence plots of the three most important explanatory variables in sample iterations of the four models (Uniform, Traffic, HabSuit, noTopo). (a) Uniform; (b) Traffic; (c) HabSuit; (d) noTopo
