# Supplementary material for: Predicting species occurrences with habitat network models
Source: Ecol Evol. 2019 Sep 4;9(18):10457–71. doi: 10.1002/ece3.5567 (PMC6787819; doi:10.1002/ece3.5567)
Supplement: Supplementary file 1 [file ECE3-9-10457-s001.docx]

**Appendix 1.** The 13 amphibian species of which presence records in the Swiss Plateau were used in this study.

| **Species** | **Common name** |  |
| --- | --- | --- |
| *Hyla arborea* | European Tree Frog |  |
| *Alytes obstetricans* | Midwife Toad |  |
| *Bombina variegata* | Yellow-bellied Toad |  |
| *Bufo bufo* | Common Toad |  |
| *Epidalea calamita* | Natterjack Toad |  |
| *Ichthyosaura alpestris* | Alpine Newt |  |
| *Lissotriton helveticus* | Palmate Newt |  |
| *Pelophylax* sp. *(P. lessonae + P. esculentus)* | Green Frog complex (Pool Frog and Edible Frog) |  |
| *Pelophylax ridibundus* | Lake Frog |  |
| *Rana dalmatina* | Agile Frog |  |
| *Rana temporaria* | Grass Frog |  |
| *Triturus carnifex* | Italian crested newt |  |
| *Triturus cristatus* | Northern crested Newt |  |
