# Supplementary material for: Predicting species occurrences with habitat network models
Source: Ecol Evol. 2019 Sep 4;9(18):10457–71. doi: 10.1002/ece3.5567 (PMC6787819; doi:10.1002/ece3.5567)
Supplement: Supplementary file 2 [file ECE3-9-10457-s002.docx]

**Appendix 2.** Final set of 20 predictor variables used in habitat suitability modelling (at a resolution of 1 ha), classified in three main categories.

| Category | Predictor | Variable type |
| --- | --- | --- |
| Human influence | Density of traffic | Continuous |
|  | Density of railways | Continuous |
|  | Total noise at nighttime | Continuous |
|  | Population density | Continuous |
|  | Agriculture density | Continuous |
|  | Arable land | Binary |
|  | Green settlements | Binary |
|  | Grey settlements | Binary |
|  | Meadows and farm pastures | Binary |
|  | Orchards, vineyards, horticulture | Binary |
| Natural landscape features | Deciduous forest coverage | Binary |
|  | Mixed forest coverage | Binary |
|  | Coniferous forest coverage | Binary |
|  | Density of forest (general) | Continuous |
|  | Distance to forest edge | Continuous |
|  | Presence of rivers | Binary |
|  | Slope | Continuous |
| Climatic variables | Mean summer precipitation | Continuous |
|  | Mean annual direct solar radiation | Continuous |
|  | Mean annual temperature | Continuous |
