# Supplementary material for: Predicting species occurrences with habitat network models
Source: Ecol Evol. 2019 Sep 4;9(18):10457–71. doi: 10.1002/ece3.5567 (PMC6787819; doi:10.1002/ece3.5567)
Supplement: Supplementary file 3 [file ECE3-9-10457-s003.docx]

**Appendix 3.** Predictor variables in habitat suitability modeling, their content and source.

| **Predictor** | **Content** | **Source** |
| --- | --- | --- |
| Density of traffic | Individual vehicle traffic for 2010 | NPVM with tunnels removed (ARE 2010) |
| Density of railways | Density of rail network | SwissTLM3D (Swisstopo 2016) |
| Total noise at nighttime | Nighttime rail noise combined with nighttime street noise | EMPA (2011) |
| Population density | Statistics on Swiss population, geolocated | STATPOP (BFS 2015) |
| Agriculture density | Density of agricultural areas, derived from an aggregate of the four main categories of agricultural land use | Arealstatistik (OFS 2010) |
| Arable land | Agricultural area taken from point estimates on 72 land use categories | Arealstatistik (OFS 2010) |
| Green settlements | Area of green spaces in settlements taken from point estimates of 72 land use categories | Arealstatistik (OFS 2010) |
| Grey settlements | Area of grey (sealed areas and buildings) areas taken from 72 land use categories | Arealstatistik (OFS 2010) |
| Meadows and pastures | Area of meadows and pastures taken from point estimates of 72 land use categories | Arealstatistik (OFS 2010) |
| Orchards, vineyards, horticulture | Area of orchards, vineyards and horticulture taken from point estimates of 72 land use | Arealstatistik (OFS 2010) |
| Deciduous forest coverage | Occurrence of deciduous forests | Waldmischungsgrad (BFS 2013) |
| Mixed forest coverage | Occurrence of mixed forests | Waldmischungsgrad (BFS 2013) |
| Coniferous forest coverage | Occurrence of coniferous forests | Waldmischungsgrad (BFS 2013) |
| Density of forest | Density of all forest types of Switzerland | Waldmischungsgrad (BFS 2013) |
| Distance to forest edge | Distance to forest edges | Waldmischungsgrad (BFS 2013) |
| Presence of rivers | Presence of rivers | SwissTLM3D (Swisstopo 2016) |
| Slope | Calculated from a digital elevation model | swissALTI3D (Swisstopo 2018) |
| Mean summer precipitation | Mean summer precipitation (1961-1990) | (Broennimann *et al.* 2003) |
| Mean annual direct solar radiation | Mean annual direct solar radiation (1961-1990) | (Broennimann *et al.* 2003) |
| Mean annual temperature | Mean annual temperature (1961-1990) | (Broennimann *et al.* 2003) |
