# Supplementary material for: Predicting species occurrences with habitat network models
Source: Ecol Evol. 2019 Sep 4;9(18):10457–71. doi: 10.1002/ece3.5567 (PMC6787819; doi:10.1002/ece3.5567)
Supplement: Supplementary file 4 [file ECE3-9-10457-s004.docx]

**Appendix 4.** Partial dependence plots of the three most important explanatory variables in sample iterations of the four models (Uniform, Traffic, HabSuit, noTopo).

1. Uniform
2. Traffic
3. HabSuit
4. noTopo
