# Supplementary figures and images for: Predicting species occurrences with habitat network models
Source: Ecol Evol. 2019 Sep 4;9(18):10457–71. doi: 10.1002/ece3.5567 (PMC6787819; doi:10.1002/ece3.5567)

Uniform

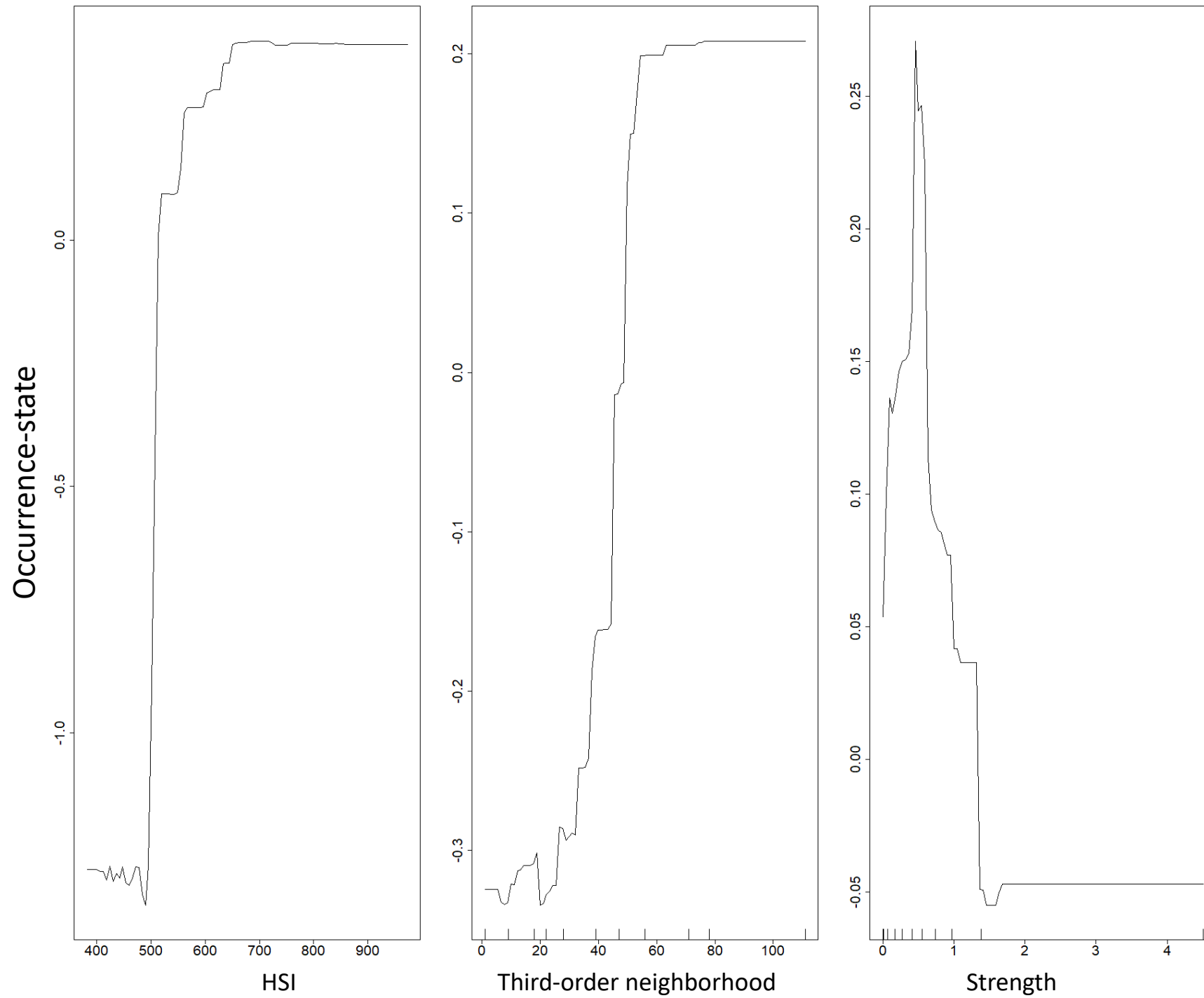

Supplement: Supplementary file 5 [file ECE3-9-10457-s005.pdf]

Traffic

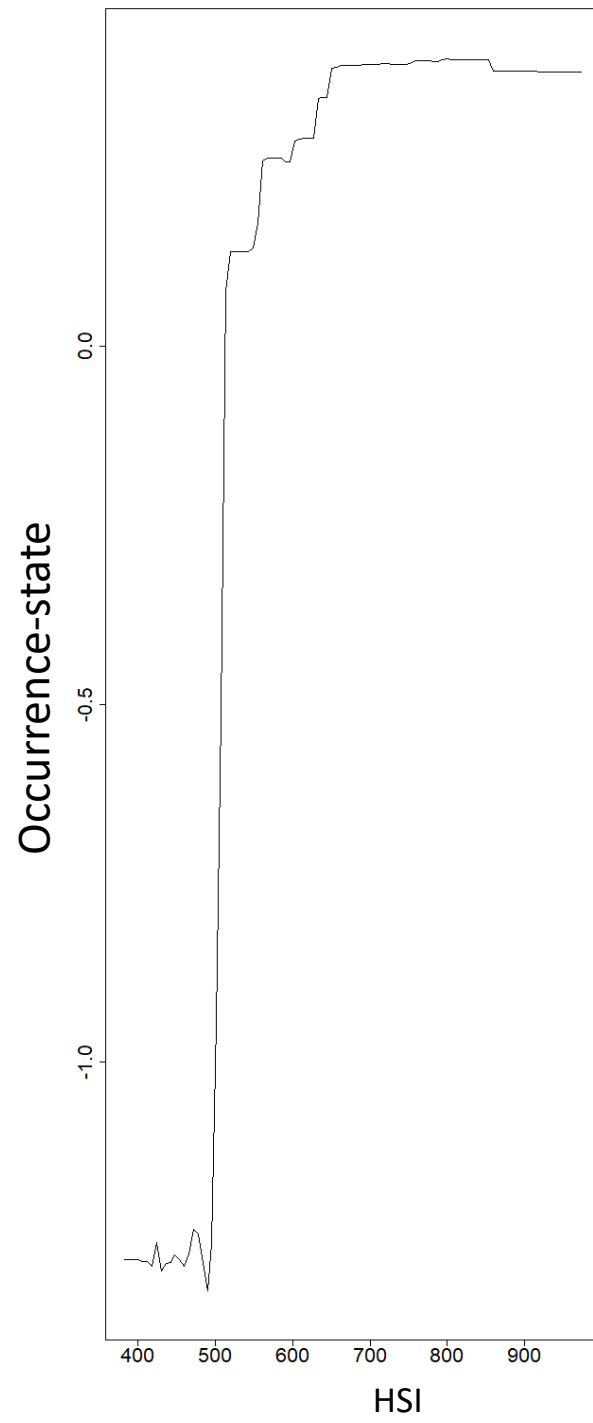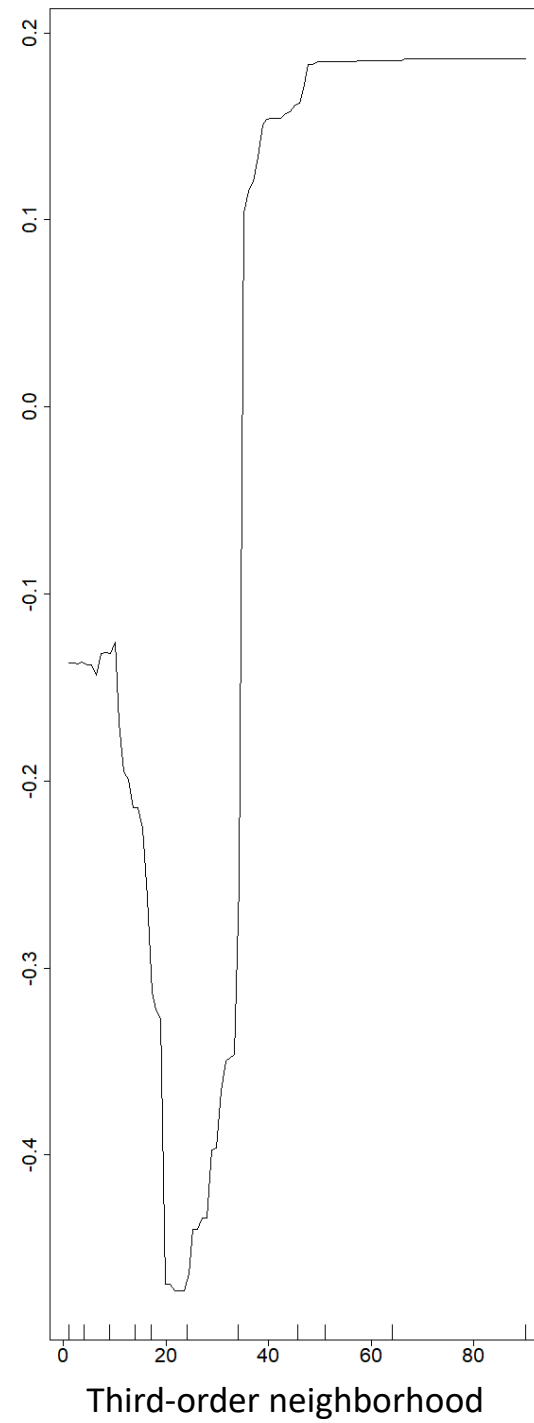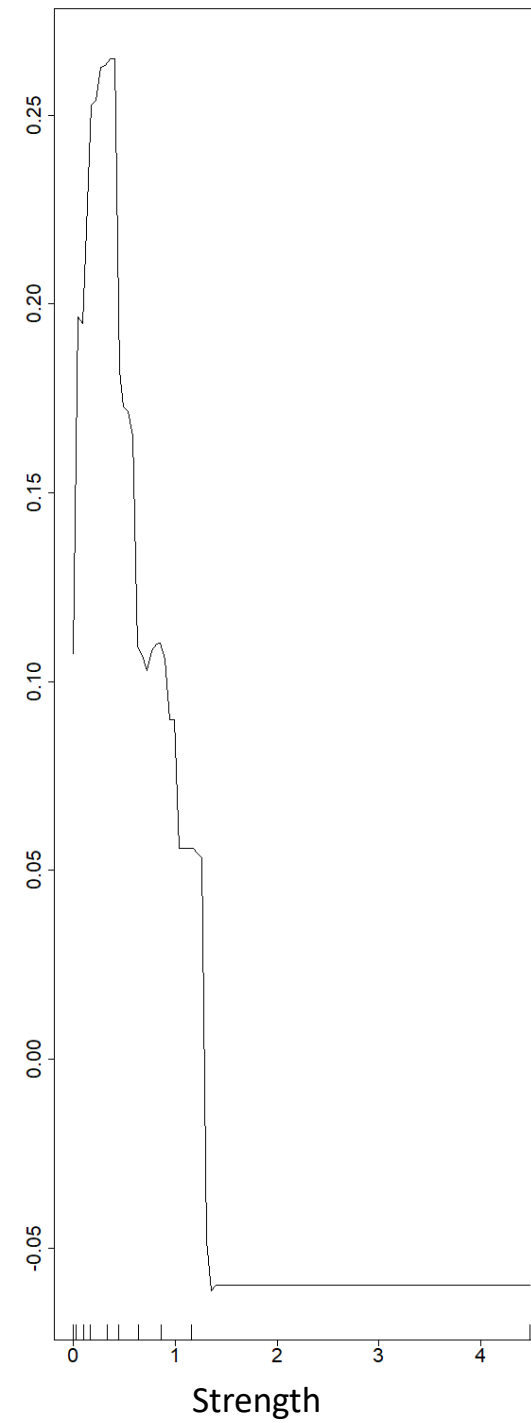

Supplement: Supplementary file 6 [file ECE3-9-10457-s006.pdf]

HabSuit

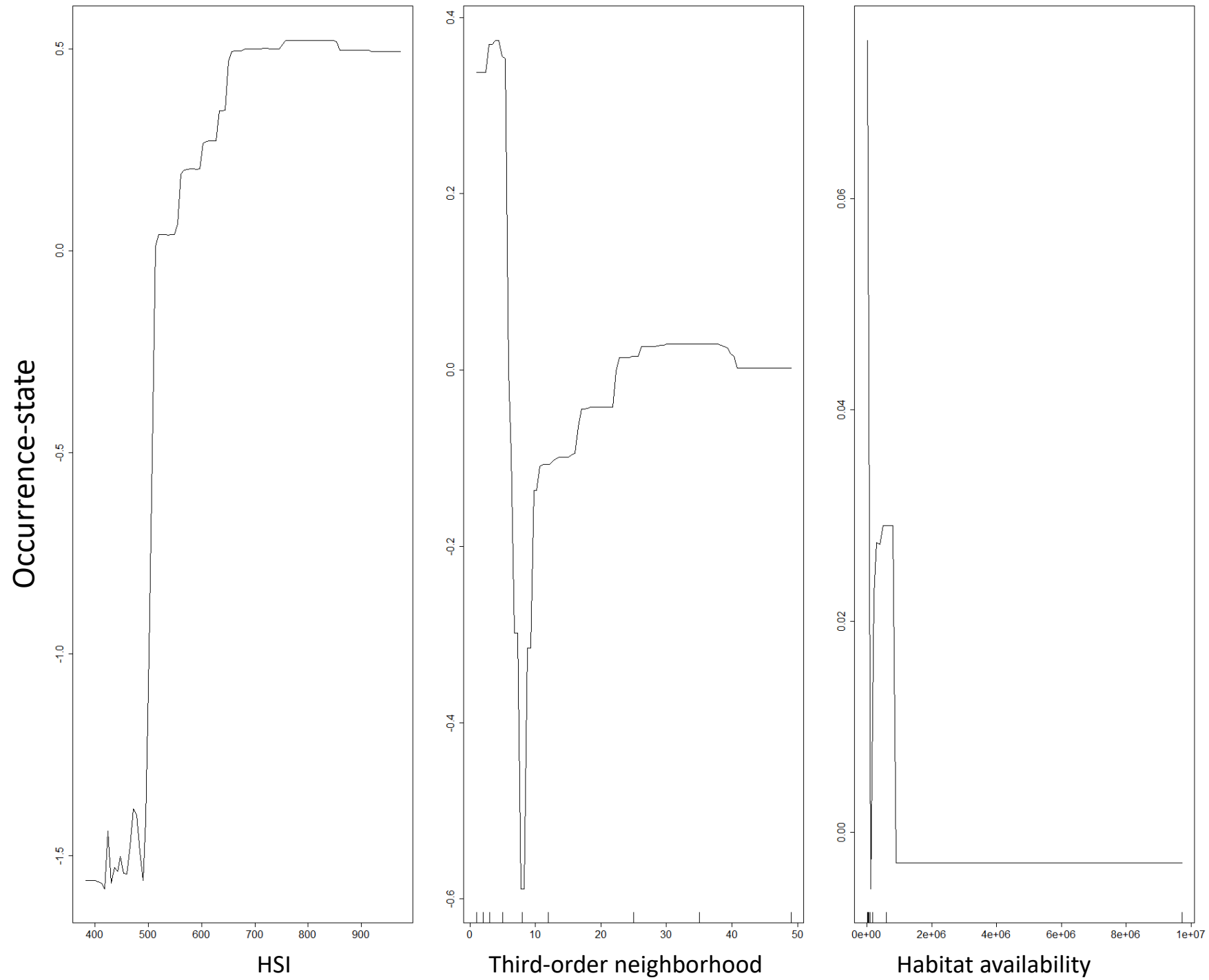

Supplement: Supplementary file 7 [file ECE3-9-10457-s007.pdf]

noTopo

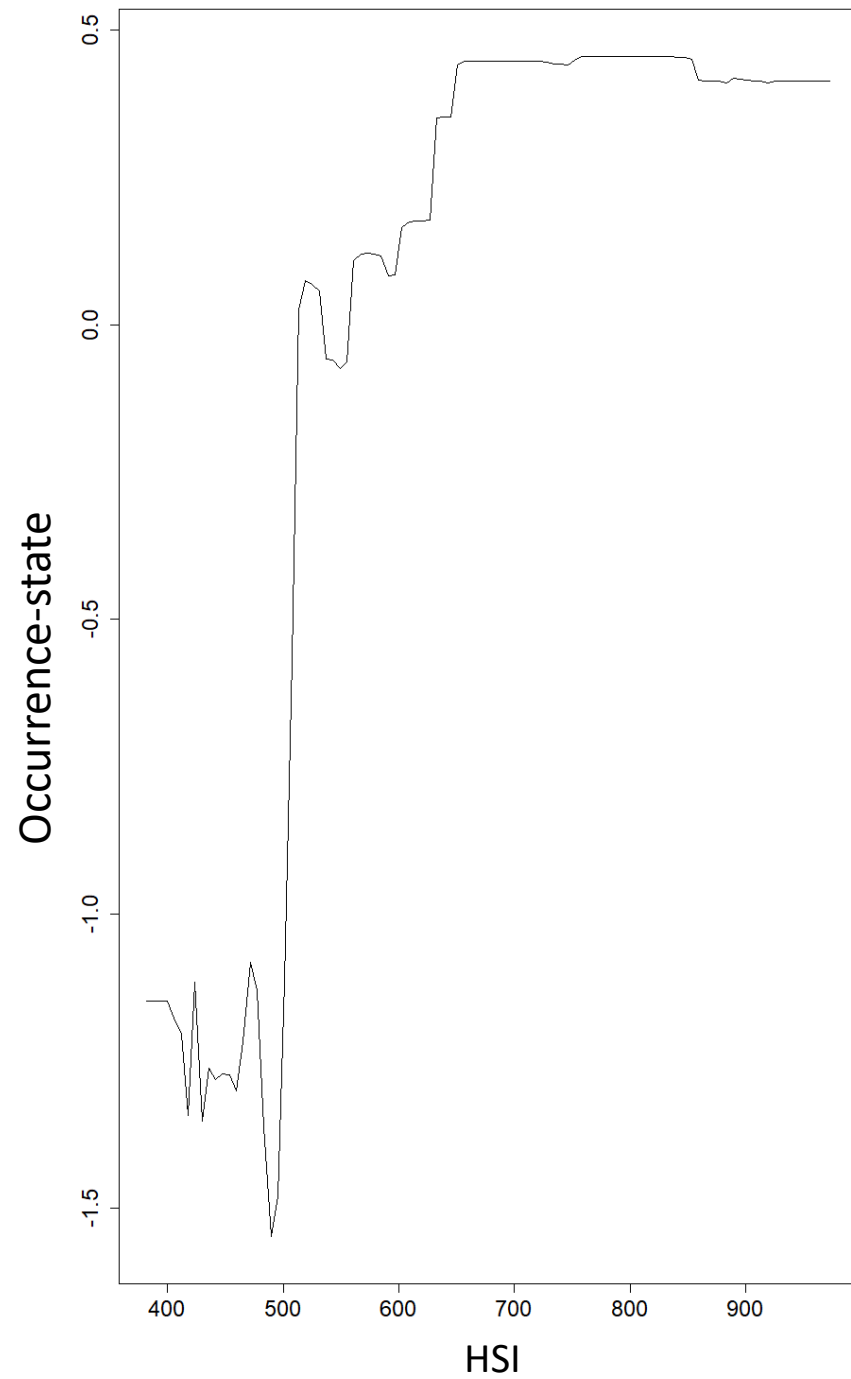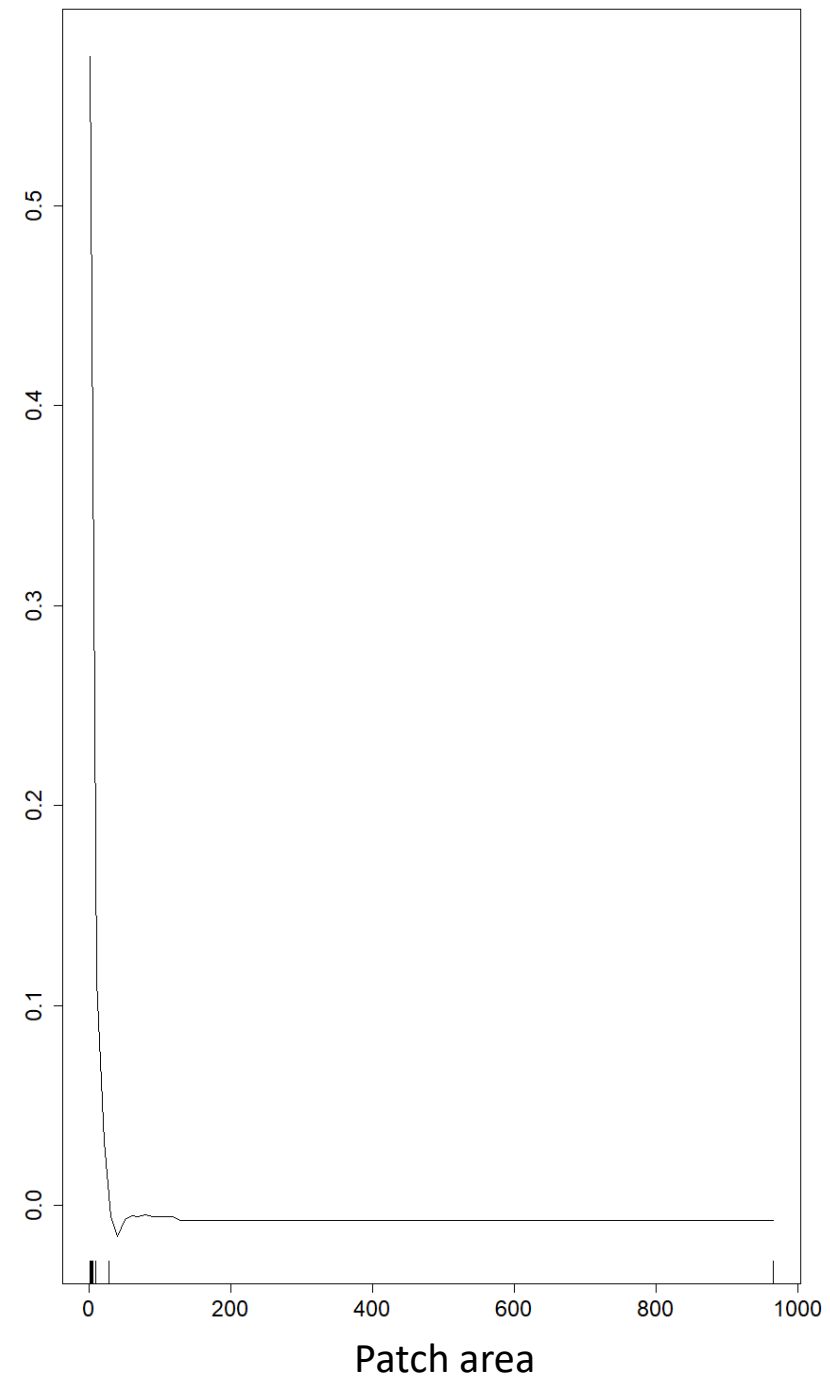

Supplement: Supplementary file 8 [file ECE3-9-10457-s008.pdf]
